# Can We Forgo the Use of Tourniquets in Total Knee Arthroplasty?

**DOI:** 10.1055/s-0044-1785204

**Published:** 2024-04-10

**Authors:** João Paulo Fernandes Guerreiro, Caio Winch Janeiro, Bruno Zarpelon, Paulo Mazzo Calzavara, Paulo Roberto Bignardi, Marcus Vinicius Danieli

**Affiliations:** 1 Faculdade de Medicina, Pontifícia Universidade Católica do Paraná – Câmpus Londrina, Londrina, Paraná, Brasil; 2 Hospital de Ortopedia Uniort.e, Londrina, Paraná, Brasil; 3 Hospital Evangélico de Londrina, Londrina, Paraná, Brasil

**Keywords:** arthroplasty, knee, hemorrhage, tourniquet

## Abstract

**Objective** To analyze whether there is more bleeding in patients undergoing total knee arthroplasty (TKA) without using a tourniquet. The secondary objectives were to analyze the operative time, the length of hospital stay, the need for transfusion, and the complication rate.

**Methods** The present is a retrospective study through the analysis of medical records. The patients were divided into two groups: TKA with and without the use of a tourniquet. Reductions in the levels of hemoglobin and packed cell volume 24 h and 48 h after surgery, the operative time, the length of hospital stay, the need for transfusion, and the rate of complications up to 6 months postoperatively were compared between the groups.

**Results** During the period analyzed, 104 patients underwent TKA, and 94 were included in the study. There were no differences between the groups regarding the mean values of hemoglobin and packed cell volume before surgery ( *p* = 0.675 and *p* = 0.265), 24 h ( *p* = 0.099 and *p* = 0.563), and 48 h ( *p* = 0.569 and *p* = 0.810) after the procedure. Neither were there differences between the groups in terms of the operative time and the length of hospital stay ( *p* = 0.484 and *p* > 0.05). Moreover, there were no differences regarding the need for transfusion and the complication rate.

**Conclusion** It is possible to forgo the use a tourniquet in TKA without a significant change in hemoglobin and packed cell volume levels 24 h and 48 h after surgery when compared with the group using a tourniquet. There were no significant differences in the total operative time, length of stay, need for transfusion, and complication rate.

## Introduction

The use of a tourniquet in total knee arthroplasty (TKA) is common, as it reportedly improves the visualization of the operative field, reduces intraoperative blood loss, and increases cementation quality. 1 2 Research carried out in 2009 at the American Association of Hip and Knee Surgeons annual meeting to analyze the medical management in TKA showed that a tourniquet was used in up to 58% of the cases. 3

However, many studies point to numerous disadvantages in adopting the tourniquet, including short-term postoperative deficits in knee flexion or extension, 1 4 5 pain and prolonged limb edema, 6 increased wound-related complications, and peripheral nerve injuries, depending on the duration of tourniquet use. 7 The tourniquet may also be associated with increased cerebral and cardiac microembolic events. 8 Some authors mention that using a tourniquet may also increase the risk of deep-vein thrombosis (DVT) 4 and skin irritation due to contact with the device. 9 The purpose of the tourniquet, supposedly to reduce perioperative blood loss, has been contested. 10 In addition, the tourniquet increases occult blood loss in certain cases.

Recent meta-analyses have demonstrated that tourniquet use is related to a greater risk of severe adverse events, postoperative pain, longer length of stay, 1 11 and increased occult bleeding. 1 The only finding potentially favoring tourniquet use would be reduced surgical time. 1 11

The primary objective of the present study was to analyze whether the postoperative reductions on the serum levels of hemoglobin (Hb) and packed cell volume (PCV) are higher in patients undergoing TKA with no tourniquet. The secondary objectives were to determine the presence of differences concerning operative time, length of stay, need for transfusion, new surgeries to drain postoperative hematoma, arthrofibrosis, wound dehiscence, superficial and deep infections, DVT, pulmonary thromboembolism (PTE), acute myocardial infarction (AMI), and deaths up to the sixth postoperative month.

We hypothesized that there would be no increase in bleeding or operative time, and that there could be a reduction in complication rates when TKA was performed without a tourniquet.

## Materials and Methods

The present study was conducted after approval form the institutional Research Ethics Committee (under number CAAE 61606122.0.0000.5696). The study followed resolution no. 466/2012 of the Brazilian National Health Council and the Declaration of Helsinki.

The present retrospective observational study analyzed medical records of patients undergoing TKA between 2017 and 2022 in the same hospital and by the same team. The patients were divided into two groups: TKA with and without a tourniquet. The levels of Hb PCV before and 24 h and 48 h after surgery, the operative time, the length of stay, the number of transfusions, new surgeries to drain hematoma in the postoperative period, arthrofibrosis, wound dehiscence, superficial and deep infections, DVT and PTE incidence rates, AMI, and deaths up to the sixth postoperative month were compared between groups.

The study included all patients over 18 years old who underwent TKA in the same hospital by the same surgical team. Patients undergoing revision TKA, those requiring associated corrective osteotomy, those with previous coagulation disorders, or those who had used an anticoagulant up to seven days before surgery were excluded.

All patients had a blood sample collected through peripheral puncture before, 24 h, and 48 h after surgery. In addition, the surgical time of each procedure was evaluated using notes from the nursing and anesthesia team. All patients underwent spinal anesthesia and surgery via the medial parapatellar approach, maintaining ischemia from the beginning of the skin incision until the final dressing in cases of tourniquet use. The implants used were cemented, subsequently stabilized, with no patellar replacement, and using the same instrumentation for implantation (Meta Bio Industrial Ltd., Rio Claro, SP, Brazil). No suction drain was used in any case. Patients operated on with a tourniquet received 1 g of tranexamic acid intra-articularly at the end of joint capsule closure. Patients not using a tourniquet received 1 g of intravenous tranexamic acid during anesthetic induction and 500 mg intra-articularly at the end of joint capsule closure. All patients received drug thromboembolic prophylaxis with 40 mg of low-molecular-weight heparin subcutaneously 6 hours after the procedure and every 24 hours during hospitalization. After hospital discharge, the patients were prescribed 10 mg of oral rivaroxaban daily for 10 days. The criterion for transfusion indication was an Hb level > 7 g/dL associated with clinical symptoms such as hemodynamic instability, oliguria, reduced level of consciousness, or regular and/or poor general condition. The criteria to indicate hospital discharge were hemodynamic stability, walking with the aid of a walker, and controlled pain.

For the statistical analysis, we considered a retrospective cohort with Hb and PCV as dependent outcomes, while age, sex, surgical time, length of stay, and complication rate were independent variables. The statistical analysis used a linear model for repeated measures with the Sidak post-hoc test. The quantitative data underwent a *t* -test, whereas the categorical variables underwent a Chi-squared test. The analysis employed the IBM SPSS Statistics for Windows (IBM Corp., Armonk, NY, United States) software, version 23.0.

## Results

**Fig. 1:**
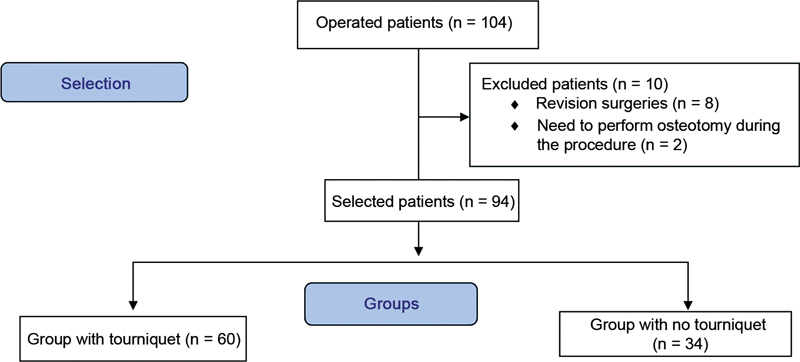
Flowchart.

During the selected period, 104 patients underwent surgery, and 94 were included in the study. Fig. 1 shows the study flowchart. Tourniquets were used in 60 patients, and 34 patients were operated on without a tourniquet. Table 1 illustrates the lack of statistical difference regarding age, sex, and initial Hb and PCV levels.

**Table 1:** Preoperative demographics

Variable	With tourniquet ( *n* = 60)	Without tourniquet ( *n* = 34)	*p* -value*
Age (years)	68.3 ± 9.13	68.2 ± 7.43	0.189
Male (%)	17 (28.3)	13 (38.2)	0.322
Hb (g/dL)	12.85 ± 1.57	13.05 ± 1.31	0.675
PCV (%)	38.21 ± 4.74	39.49 ± 4.19	0.265

**Abbreviations:** Hb, hemoglobin; PCV, packed cell volume.

**Note:** Quantitative data are represented as mean ± standard deviation values.

Table 2 shows that although the mean surgical time in the group not using a tourniquet was higher, this difference was not statistically significant ( *p* = 0.484). The length of stay in days was not different between the groups ( *p* > 0.05).

**Table 2:** Comparison between groups regarding surgical time and length of stay

Variable	With tourniquet ( *n* = 60)	With no tourniquet ( *n* = 34)	*p* -value*
Surgical time (minutes)	107.16 ± 2.94	111.79 ± 26.17	0.484
Length of stay (days)	2.07 ± 0.25	2.15 ± 0.36	> 0.05

**Note:** Data are represented as mean ± standard deviation values.

Table 3 presents the complication rates. There was no difference between groups in any of the variables analyzed. Moreover, there were no reports of PTE or surgery for hematoma drainage.

**Table 3:** Postoperative complications in each group

Variable	With tourniquet ( *n* = 60)	Without tourniquet ( *n* = 34)	*p* -value*
Need for transfusion	1	0	> 0.05
Superficial infection	4	1	> 0.05
Deep infection	1	1	> 0.05
Arthrofibrosis management	1	1	> 0.05
Deep-vein thrombosis	1	1	> 0.05
Acute myocardial infarction	2	0	> 0.05
Death	2	0	> 0.05
TOTAL	12	4	> 0.05

**Fig. 2:**
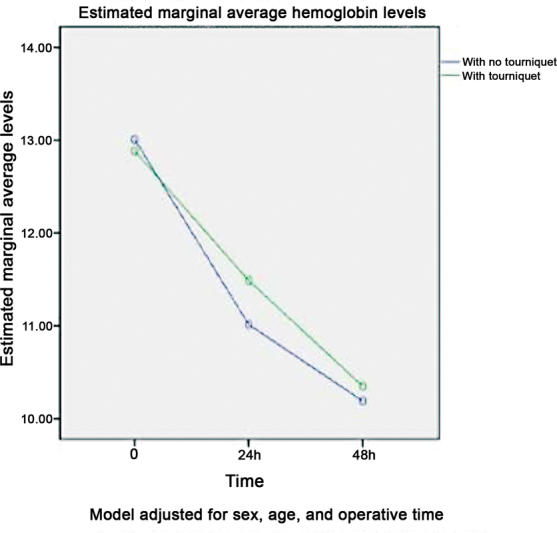
Variation in hemoglobin levels in patients undergoing total knee arthroplasty.

**Fig. 3:**
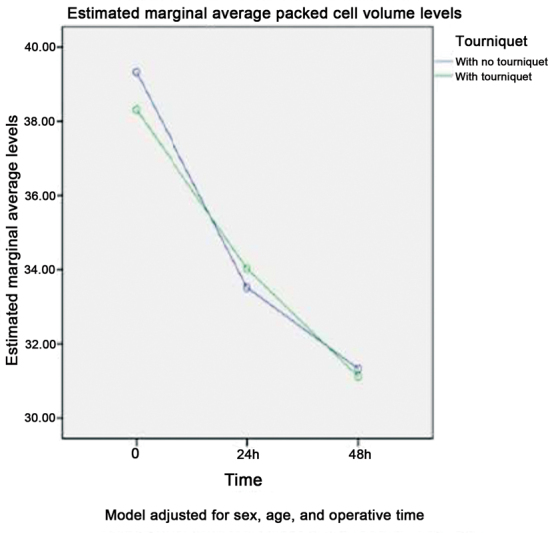
Variation in packed cell volume levels in patients undergoing total knee arthroplasty.

Fig. 2 shows the mean Hb values in both groups before surgery (95% confidence interval [95%CI]: -0.711–0.492; *p* = 0.675) and 24 h (95%CI: -0.091–1.039; *p* = 0.099) and 48 h (95%CI: -0.391–0.706; *p* = 0.569) after the procedure. Fig. 3 shows the mean PCV levels in both groups before surgery (95%CI: -2.814–0.783; *p* = 0.265) and 24 h (95%CI: -1.238–2.260; *p* = 0.563) and 48 h (95%CI: -1.944–1.522; *p* = 0.810) after the procedure.

## Discussion

The present study showed no significant difference in terms of the postoperative reductions on the levels of Hb and PCV, a finding similar to those of meta-analyses published in 2014 4 and 2021, 11 but contrary to those of meta-analyses published in 2019 10 and 2022. 1 The difference in Hb loss in the first 24 h between groups was the most significant result, but it was not significant ( *p* = 0.099). In addition, after the first 48 h, the Hb levels were similar between the groups ( *p* = 0.569). We suppose this initial trend of a higher drop in Hb levels in the group not using a tourniquet may have occurred due to earlier bleeding. This drop would be detectable in tests performed within the first 24 h, which may not occur in the group using the tourniquet, which would theoretically have a later, hidden bleeding. This detail may justify the divergence in the literature on this subject. 4 10 11 Sun et al. 1 showed less intraoperative bleeding and more occult bleeding in the tourniquet group. This paper 1 only included studies using tranexamic acid, which was also a routine in our patients and has proven effective in bleeding reduction. 12

The operative time was not significantly different between the groups. The mean surgical time was of 107.16 ± 22.94 and 111.79 ± 26.17 minutes in the groups using or not using tourniquets respectively ( *p* = 0.484). This finding differs from those of the latest meta-analyses indicating that the tourniquet would contribute to reducing the operative time. 1 11 We believe including more patients in the study could show some difference concerning this variable. Alternatively, the experience of our group in operating without a tourniquet may have approximated the surgical times in both modalities.

The potential postoperative complications reported in the literature, 5 11 13 such as increased length of stay and need for transfusion, new surgeries for hematoma drainage in the postoperative period and for arthrofibrosis, wound dehiscence, superficial and deep infections, DVT, PTE, AMI, and deaths. The rates of these complications were not different between the two groups.

In a prospective study with more than 150 patients published in 2021, Zak et al. 14 reported that the length of stay was equal between the groups. Yi et al., 5 in 2021, demonstrated, in a prospective study with 50 patients in each group, a lower length of stay in the group not using a tourniquet. In the present study, the length of stay was not different between the groups using a tourniquet or not (2.07 ± 0.25 and 2.15 ± 0.36 respectively; *p* > 0.05). Both studies 5 14 cited for comparison used tranexamic acid during surgery and did not mention their criteria for hospital discharge, which in the study included hemodynamic stability, the ability to walk with the aid of a walker, and pain control.

A single patient from the tourniquet group required a blood transfusion. However, this subject had preoperative chronic anemia, and the drop in Hb and PCV levels was within the standard observed in other patients. In a prospective study published in 2019, Alexandersson et al. 13 reported 1 transfusion in the group not using a tourniquet (consisting of 43 patients) and 4 transfusions in the group with a tourniquet (38 patients). The same study 13 reported two cases of infections in the group using a tourniquet (one superficial infection and one deep infection) and no infection in the group without a tourniquet. These data are consistent with those of the present study, since we observed higher numbers of infections in the group with a tourniquet despite the lack of statistical difference between them (four cases of superficial infections and one deep infection in the group with a tourniquet, and one case of superficial infection and one deep infection in the group without a tourniquet).

We did not observe cases of clinical DVT in the present study, but we did not actively search for thrombi with imaging tests. A previous prospective study 15 with groups of 60 patients receiving tranexamic acid during the procedure did not find any case of clinical DVT; however, the tourniquet group presented more cases of asymptomatic thrombi at an ultrasound examination of the lower limbs. Large retrospective studies 16 17 demonstrated a low rate of clinical DVT after TKA with and without a tourniquet. In one of them, using the Danish registry 16 with 19,804 patients, the clinical DVT rate was low in both groups, with no difference between them (0.77% in the tourniquet group and 1.10% in the group not using a tourniquet).

The present study has limitations. Firstly, it is a retrospective study. A prospective randomized study could lead to different results. Secondly, the use of data from a single center limits the number of patients studied despite reducing the bias of comparing different surgeons with different practices. Thirdly, the number of patients was low for a retrospective study. And fourthly, we do not have data on functional and pain assessments to compare the groups.

Our results were not in line with those of the current literature showing that, with the use of tranexamic acid and tourniquets in an optimized and customized way 1 2 18 during TKA, the advantages of the tourniquet are obtained without increasing the complication rates.

## Conclusion

It is possible not to use a tourniquet in TKA without significant changes in Hb and PCV levels 24 h and 48 h after surgery compared with the group using a tourniquet. Likewise, there was no significant difference in operative time, length of stay, need for transfusion, new surgeries for postoperative hematoma drainage and arthrofibrosis, wound dehiscence, superficial and deep infections, DVT, PTE, AMI, and deaths between the two groups.
