# Supplementary material for: Can We Forgo the Use of Tourniquets in Total Knee Arthroplasty?
Source: Rev Bras Ortop (Sao Paulo). 2024 Apr 10;59(2):e235–40. doi: 10.1055/s-0044-1785204 (PMC11006514; doi:10.1055/s-0044-1785204)
Supplement: Related texts

# Research article: Podemos dispensar o uso do torniquete nas artroplastias totais do joelho?

**Keywords:** artroplastia, joelho, hemorragia, torniquete

## Abstract

**Objetivo** Analisar se há mais sangramento em pacientes submetidos a artroplastia total do joelho (ATJ) sem a utilização do torniquete. Os objetivos secundários foram analisar os tempos cirúrgico e de internação, a necessidade de transfusão e a taxa de complicações.

**Métodos** Estudo retrospectivo por meio da análise de prontuários. Os pacientes foram divididos em dois grupos: ATJ com e sem a utilização de torniquete. Foram comparados os valores das quedas da hemoglobina e do hematócrito 24 h e 48 h após a cirurgia, os tempos cirúrgico e de internação, a necessidade de transfusão e a taxa de complicações até 5 meses de pós-operatório.

**Resultados** No período, foram operados 104 pacientes, sendo 94 incluídos no estudo. Não houve diferença entre as médias dos valores da hemoglobina e do hematócrito entre os grupos no pré-operatório ( *p* = 0,675 e *p* = 0,265, respectivamente), 24 h ( *p* = 0,099 e *p* = 0,563, respectivamente) e 48 h ( *p* = 0,569 e *p* = 0,810, respectivamente) após a cirurgia. O tempo cirúrgico e o tempo de internação também não foram diferentes entre os grupos ( *p* = 0,484 e *p* > 0,05, respectivamente). Não houve diferença com relação à necessidade de transfusões, nem com relação à taxa de complicações analisadas.

**Conclusão** Podemos dispensar o uso do torniquete nas cirurgias de ATJ sem alteração significativa no valor dos níveis de hemoglobina e de hematócrito 24 h e 48 h após a cirurgia quando comparado ao grupo com uso de torniquete. Não houve diferenças significativas no tempo total das cirurgias, no tempo de internação, na necessidade de transfusões e nas taxas de complicações analisadas.

## Introdução

O uso de torniquete na cirurgia de artroplastia total de joelho (ATJ) é frequente, pois acredita-se que melhora a visualização do campo operatório, diminui a perda intraoperatória de sangue e melhora a qualidade da cimentação. 1 2 Uma pesquisa realizada em 2009, 3 na reunião anual da American Association of Hip and Knee Surgeons, para analisar condutas médicas em cirurgias de ATJ, mostrou que o torniquete era utilizado em até 58% dos casos.

Contudo, muitos estudos apontam para inúmeras desvantagens na adoção desse instrumento, como déficit de flexão ou de extensão do joelho no pós-operatório em curto prazo, 1 4 5 dor e edema prolongado do membro, 6 aumento das complicações relacionadas à ferida e lesões de nervos periféricos conforme o tempo de utilização do torniquete. 7 O torniquete também pode estar associado ao aumento de eventos microembólicos cerebrais e cardíacos. 8 Alguns autores afirmam que o uso de torniquete também pode estar relacionado ao aumento do risco de tromboembolismo venoso profundo (TVP) 4 e de irritação da pele pelo contato com o torniquete. 9 O propósito do torniquete, que deveria ser a redução da perda sanguínea perioperatória, já foi contestado, 10 e mostrou-se que em certos casos há aumento da perda sanguínea oculta. 1 10

Metanálises recentes demonstraram que o uso de torniquete está relacionado a um maior risco de eventos adversos graves, dor pós-operatória, maior tempo de hospitalização 1 11 e mais sangramento oculto. 1 O único achado a favorecer o uso do torniquete seria uma redução no tempo de cirurgia. 1 11

O objetivo primário deste estudo foi analisar se há uma queda maior nos valores da hemoglobina (Hb) e do hematócrito (Ht) séricos no pós-operatório em pacientes submetidos à cirurgia de ATJ sem a utilização do torniquete. Os objetivos secundários foram analisar se houve diferença em relação ao tempo cirúrgico, aos dias de internação, à necessidade de transfusões, a novas cirurgias para drenagem de hematoma no pós-operatório, à artrofibrose, à deiscência de ferida, às infecções superficial e profunda, à TVP e ao tromboembolismo pulmonar (TEP), ao infarto agudo do miocárdio (IAM) e a óbitos até o sexto mês de pós-operatório.

A nossa hipótese era a de que não haveria aumento no sangramento, nem no tempo cirúrgico, e que poderia haver uma redução nas taxas de complicações quando a ATJ fosse realizada sem o torniquete.

## Materiais e Métodos

O presente estudo foi realizado após aprovação do Comitê de Ética em Pesquisa institucional (CAAE 61606122.0.0000.5696) de acordo com a resolução n° 466/2012 do Conselho Nacional de Saúde e a Declaração de Helsinque.

Foi feito um estudo observacional retrospectivo pela análise dos prontuários de pacientes submetidos a ATJ entre 2017 e 2022 no mesmo hospital e pela mesma equipe. Os pacientes foram divididos em dois grupos: ATJ com e sem o uso de torniquete. Foram comparados os valores de Hb e Ht pré-operatórios e 24 h e 48 h após a operação, o tempo cirúrgico, os dias de internação, o número de transfusões, as novas cirurgias para drenagem de hematoma no pós-operatório, o tratamento de artrofibrose, a deiscência de ferida, as infecções superficial e profunda, as incidências de TVP e TEP, o IAM e os óbitos até o sexto mês de pós-operatório.

Foram incluídos todos os pacientes maiores de 18 anos que foram submetidos a ATJ no mesmo hospital e pela mesma equipe cirúrgica. Foram excluídos os pacientes submetidos a ATJ de revisão, aqueles com necessidade de osteotomia corretiva associada, pacientes com distúrbios de coagulação prévios, e aqueles que tinham feito uso de algum anticoagulante até sete dias antes da cirurgia.

Todos os pacientes tiveram amostra de sangue coletada por meio de punção periférica no pré-operatório, e 24 h e 48 horas após a cirurgia. Além disso, foi avaliado o tempo cirúrgico de cada procedimento pelas anotações da equipe de enfermagem e de anestesia. Todos os pacientes foram submetidos a raquianestesia, operados pela via de acesso parapatelar medial, mantendo a isquemia desde o início da incisão da pele até o curativo final nos casos de uso de torniquete. Os implantes utilizados foram sempre cimentados, com estabilização posterior, sem a substituição da patela e utilizando o mesmo instrumental para a implantação (Meta Bio Industrial Ltda., Rio Claro, SP, Brazil). Não foi utilizado dreno de sucção em nenhum caso. Nos pacientes operados com torniquete, 1 g de ácido tranexâmico foi aplicado de modo intra-articular ao final do fechamento da cápsula articular. Nos pacientes sem o uso de torniquete, 1 g de ácido tranexâmico endovenoso foi administrado na indução anestésica e 500 mg de modo intra-articular ao final do fechamento da capsula articular. Profilaxia tromboembólica medicamentosa foi realizada em todos os pacientes, com 40 mg de heparina de baixo peso molecular por via subcutânea após 6 h do procedimento, e depois foi mantida a mesma dose a cada 24 h durante e internação. Após a alta hospitalar, 10 mg diárias de rivaroxabana por via oral foram prescritas por 10 dias. O critério para indicar a transfusão foi Hb > 7 g/dL associada a sintomas clínicos, como instabilidade hemodinâmica, oliguria, rebaixamento do nível de consciência ou regular e/ou mal estado geral. Os critérios para indicar a alta hospitalar foram estabilidade hemodinâmica, caminhar com auxílio de andador e dor controlada.

Na análise estatística, foi considerada uma coorte retrospectiva com Hb e Ht como desfechos dependentes, e idade, sexo, tempo de cirurgia, dias de internação e taxa de complicações como variáveis independentes, que foram analisados pelo modelo linear para medidas repetidas com o teste *post hoc* de Sidak. Para os dados quantitativos, a análise foi realizada por meio do teste *t* . Para as variáveis categóricas foi utilizado o teste do qui-quadrado. Foi utilizado o programa IBM SPSS Statistics for Windows (IBM Corp., Armonk, NY, Estados Unidos), versão 23.0.

## Resultados

No período selecionado, foram operados 104 pacientes, sendo 94 incluídos no estudo. Na Fig. 1 é apresentado o fluxograma do estudo. O grupo com uso do torniquete totalizou 60 pacientes, e o sem torniquete, 34 pacientes. Na Tabela 1 observa-se que não houve diferença estatística entre a idade, sexo, e Hb e Ht iniciais.

**Fig. 1:** Fluxograma.

**Tabela 1:** Dados demográficos pré-operatórios

Variável	Torniquete ( *n* = 60)	Sem torniquete ( *n* = 34)	Valor de *p* *
Idade (anos)	68,3 ± 9,13	68,2 ± 7,43	0,189
Sexo masculino (%)	17 (28,3)	13 (38,2)	0,322
Hb (g/dL)	12,85 ± 1,57	13,05 ± 1,31	0,675
Ht (%)	38,21 ± 4,74	39,49 ± 4,19	0,265

**Abreviaturas:** Hb, hemoglobina; Ht, hematócrito.

**Nota:** Os dados quantitativos apresentados por valores de média ± desvio padrão.

Na Tabela 2 pode ser observado que, apesar de o tempo cirúrgico no grupo sem torniquete ter uma média maior, tal diferença não foi estatisticamente significativa ( *p* = 0,484). Quanto ao tempo de internação em dias, tampouco houve diferença entre os grupos ( *p* > 0,05).

**Tabela 2:** Comparação entre os grupos com relação aos tempos de cirurgia e de internação

Variável	Torniquete ( *n* = 60)	Sem torniquete ( *n* = 34)	Valor de *p* *
Duração da cirurgia (min)	107,16 ± 22,94	111,79 ± 26,17	0,484
Tempo de internação (dias)	2,07 ± 0,25	2,15 ± 0,36	> 0,05

**Nota:** Dados apresentados por valores de média ± desvio padrão.

A Tabela 3 apresenta as taxas de complicações. Não houve diferença entre os grupos em nenhuma das variáveis analisadas. No estudo não foram identificados casos de TEP ou de cirurgia para drenagem de hematoma.

**Tabela 3:** Complicações pós cirúrgicas de cada grupo

Variável	Torniquete ( *n* = 60)	Sem torniquete ( *n* = 34)	Valor de *p* *
Necessidade de transfusão	1	0	> 0,05
Infecção superficial	4	1	> 0,05
Infecção profunda	1	1	> 0,05
Manipulação por artrofibrose	1	1	> 0,05
Tromboembolismo venoso profundo	1	1	> 0,05
Infarto agudo do miocárdio	2	0	> 0,05
Óbito	2	0	> 0,05
TOTAL	12	4	> 0,05

A Fig. 2 mostra as médias dos valores da Hb nos grupos, no pré-operatório (intervalo de confiança de 95% [IC95%]: −0,711–0,492; *p* = 0,675), 24 h (IC95%: −0,091–1,039; *p* = 0,099) e 48 h (IC95%: −0,391–0,706; *p* = 0,569) após o procedimento. A Fig. 3 , da mesma maneira, mostra as médias dos valores do Ht nos grupos, no pré-operatório (IC95%: −2,814–0,783; *p* = 0,265), 24 h (IC95%: −1,238–2,260 e p 0,563) e 48 h (IC95%: −1,944–1,522; *p* = 0,810) após a cirurgia.

**Fig. 2:** Variação dos valores de hemoglobina de pacientes submetidos a artroplastia total do joelho.

**Fig. 3:** Variação dos valores de hematócrito de pacientes submetidos a artroplastia total de joelho.

## Discussão

Neste estudo, não houve diferença significativa entre os grupos com relação à queda dos níveis da Hb e do Ht no pós-operatório, resultado similar ao de metanálises publicadas em 2014 4 e 2021, 11 mas contrário ao de metanálises publicadas em 2019 10 e 2022. 1 A diferença de perda de Hb nas primeiras 24 h entre os grupos foi o resultado mais expressivo, mas não foi significativo ( *p* = 0,099), e após as primeiras 48 h os valores da Hb nos grupos foram mais similares ( *p* = 0,569). Supõe-se que essa tendência inicial de maior queda nos valores da Hb no grupo sem torniquete possa ter ocorrido devido ao fato de o sangramento acontecer de forma mais precoce. Ele já seria bem detectado nos exames das primeiras 24 h, o que pode não acontecer no grupo com uso do torniquete, que teoricamente teria um sangramento mais tardio, oculto. Esse detalhe pode justificar a divergência na literatura sobre esse assunto, 4 10 11 e pode ser explicado pela forma como é apresentada por Sun et al., 1 quando é mostrado menos sangramento no intraoperatório e mais sangramento oculto no grupo com torniquete. Nesse estudo, 1 foram incluídos apenas trabalhos com o uso de ácido tranexâmico, que também foi a rotina nos pacientes aqui avaliados, e que já tem eficácia em reduzir o sangramento bem demonstrada. 12

Um dado que também não apresentou diferença significativa entre os grupos foi o tempo cirúrgico. A média variou entre 107,16(± 22,94) e 111,79(± 26,17) minutos entre os grupos com e sem uso de torniquete, respectivamente ( *p* = 0,484). Esse achado difere dos das últimas metanálises, 1 11 que indicam que o torniquete seria um fator de redução de tempo cirúrgico. Entendemos que a inclusão de um maior número de pacientes no estudo poderia talvez mostrar alguma diferença com relação a esta variável, ou que a experiência do nosso grupo em operar sem garrote já aproximou os tempos nas duas modalidades.

As possíveis complicações pós-operatórias já relatadas na literatura, 5 11 13 como aumento nos dias de internação e no número de transfusões, novas cirurgias para a drenagem de hematoma no pós-operatório e para a liberação de artrofibrose, deiscência de ferida, infecções superficial e profunda, TVP, TEP, IAM e óbitos, não se mostraram diferentes entre os dois grupos.

Em um estudo prospectivo com mais de 150 pacientes publicado em 2021, Zak et al. 14 mostraram que o tempo de internação foi igual entre os grupos. Yi et al., 5 também em 2021, demonstraram, em um estudo prospectivo com 50 pacientes em cada grupo, menos dias de internação no grupo sem torniquete. Neste estudo, o tempo de internação não se mostrou diferente entre os grupos com e sem torniquete (2,07 ± 0,25 e 2,15 ± 0,36, respectivamente; *p* > 0,05). Ambos estudos 5 14 citados para comparação utilizaram ácido tranexâmico durante a cirurgia e não citam os critérios utilizados para alta hospitalar, que, neste estudo, foi dada aos pacientes com estabilidade hemodinâmica, capazes de caminhar com auxílio de andador e com a dor controlada.

Apenas um paciente necessitou de transfusão sanguínea no grupo com torniquete, mas ele apresentava anemia crônica pré-operatória, e a queda nos valores da Hb e do Ht ficaram dentro do padrão dos outros pacientes. Num estudo prospectivo publicado em 2019, Alexandersson et al. 13 apresentaram 1 caso de transfusão no grupo sem torniquete (que tinha 43 pacientes) e 4 transfusões no grupo com torniquete (que tinha 38 pacientes). Ainda nesse estudo, 13 houve dois casos de infecções no grupo com torniquete (um deles foi considerada infecção superficial e o outro, profunda), e nenhum caso de infecção no grupo sem torniquete. Esse dado é similar ao deste estudo, em que foram observados mais casos de infecção no grupo com torniquete, apesar de não haver diferença estatística entre eles (4 casos de infecções superficiais e 1 profunda no grupo com torniquete, e 1 caso de infecção superficial e 1 profunda no grupo sem torniquete).

Não houve casos de TVP clínica neste estudo, mas não fizemos a busca ativa de trombos com exames de imagem. Um estudo prospectivo prévio 15 com grupos de 60 pacientes, no qual também foi utilizado o ácido tranexâmico no procedimento, não encontrou TVP clínica em nenhum caso, mas no grupo com torniquete foram encontrados, mediante exame de ultrassonografia dos membros inferiores, mais casos de trombos assintomáticos. Grandes estudos retrospectivos 16 17 já demonstraram baixo índice de TVP clínica após a ATJ com e sem torniquete. Em um deles, utilizando o registro dinamarquês, 16 com 19.804 pacientes, o índice de TVP clínico foi baixo em ambos os grupos, e sem diferença entre eles (0,77% no grupo com torniquete e 1,10% no grupo com torniquete).

Este estudo apresenta limitações. Primeiro, por ser um estudo retrospectivo. Um estudo prospectivo randomizado talvez levasse a resultados diferentes. Segundo, por utilizar dados de um único centro, o que limita a quantidade de pacientes estudados, apesar de reduzir o viés de comparar diferentes cirurgiões, com práticas diferentes. Terceiro, pelo número limitado de pacientes para um estudo retrospectivo. E quarto, por não termos dados sobre avaliações funcionais e de dor para comparar os grupos.

Ao final do estudo, foram observados resultados que vão de encontro com os da literatura mais atual, que tem mostrado que, com o uso do ácido tranexâmico e do torniquete de forma otimizada e personalizada, 1 2 18 durante a cirurgia de ATJ, obtêm-se as vantagens do torniquete sem aumentar a taxa de complicações.

## Conclusão

Podemos dispensar o uso do torniquete nas cirurgias de ATJ sem alteração significativa nos níveis de Hb e Ht 24 h e 48 h após a cirurgia, quando comparado ao grupo com uso de torniquete. Não houve diferença significativa no tempo cirúrgico, no tempo de internação, na necessidade de transfusões, nas novas cirurgias para drenagem de hematoma no pós-operatório e para a liberação de artrofibrose, na deiscência de ferida, nas infecções superficial e profunda, no TVP, no TEP, no IAM e óbitos entre os dois grupos.
